# Composition, structure, and functional shifts of prokaryotic communities in response to co-composting of various nitrogenous green feedstocks

**DOI:** 10.1186/s12866-023-02798-w

**Published:** 2023-03-02

**Authors:** Felix Matheri, Anne Kelly Kambura, Maina Mwangi, Nehemiah Ongeso, Edward Karanja, Noah Adamtey, Elias Kihara Mwangi, Edwin Mwangi, Chrysantus Tanga, Martha Wangu Musyoka, Steven Runo

**Affiliations:** 1 International Centre for Insect Physiology and Ecology (icipe), P.O. Box 30772 – 00100, Nairobi, Kenya; 2 Kenyatta University (KU), P.O. Box 43844 – 00100, Nairobi, Kenya; 3 Taita Taveta University (TTU), P.O. Box 635 – 80300, Voi, Kenya; 4 The University of Nairobi (UON), P.O. Box 30197, GPO, Nairobi, Kenya; 5 International Division, Research Institute of Organic Agriculture (FiBL), Ackerstrasse 775, CH-5070 Frick, Switzerland; 6 Biofarms (K) Limited, P.O. Box 7277 - 01000, Thika, Kenya

**Keywords:** Microbe-microbe interactions, Lantana, Tithonia, Grass, Organic farming, Composting

## Abstract

**Background:**

Thermophilic composting is a promising method of sanitizing pathogens in manure and a source of agriculturally important thermostable enzymes and microorganisms from organic wastes. Despite the extensive studies on compost prokaryotes, shifts in microbial profiles under the influence of various green materials and composting days are still not well understood, considering the complexity of the green material sources. Here, the effect of regimens of green composting material on the diversity, abundance, and metabolic capacity of prokaryotic communities in a thermophilic compost environment was examined.

**Methods:**

Total community 16S rRNA was recovered from triplicate compost samples of Lantana-based, Tithonia-based, Grass-based, and mixed (Lantana + Tithonia + Grass)- based at 21, 42, 63, and 84 days of composting. The 16S rRNA was sequenced using the Illumina Miseq platform. Bioinformatics analysis was done using Divisive Amplicon Denoising Algorithm version 2 (DADA2) R version 4.1 and Phylogenetic Investigation of Communities by Reconstruction of Unobserved States version 2 (PICRUSt2) pipelines for community structure and metabolic profiles, respectively. In DADA2, prokaryotic classification was done using the Refseq-ribosomal database project (RDP) and SILVA version 138 databases.

**Results:**

Our results showed apparent differences in prokaryotic community structure for total diversity and abundance within the four compost regimens and composting days. The study showed that the most prevalent phyla during composting included *Acidobacteriota*, *Actinobacteriota*, *Bacteroidota*, *Chloroflexi*, and *Proteobacteria*. Additionally, there were differences in the overall diversity of metabolic pathways but no significant differences among the various compost treatments on major metabolic pathways like carbohydrate biosynthesis, carbohydrate degradation, and nitrogen biosynthesis.

**Conclusion:**

Various sources of green material affect the succession of compost nutrients and prokaryotic communities. The similarity of amounts of nutrients, such as total Nitrogen, at the end of the composting process, despite differences in feedstock material, indicates a significant influence of composting days on the stability of nutrients during composting.

**Supplementary Information:**

The online version contains supplementary material available at 10.1186/s12866-023-02798-w.

## Introduction

The release of unprocessed organic waste from municipal, livestock manure, kitchen, and crop residues, to the environment poses imminent risks of spreading animal and plant pathogens [1, 2]. Managing such wastes through composting processes is essential, leading to a valuable soil resource [3]. Composting has been principally categorized as aerobic/thermophilic/hot or anaerobic [1]. Newer approaches, such as vermicomposting and the use of insects such as the black soldier fly, are gaining acceptance [4]. Nonetheless, thermophilic composting remains the method of choice since it is faster than other composting methods. Moreover, thermophilic composting can sanitize pathogens borne in the feedstock materials. This is because these pathogens cannot withstand the high temperatures of thermophilic compost [5].

Naturally, thermophilic compost piles gradually heat up to temperatures above 50 °C, followed by sustained high temperatures above 60 °C and a final gradual heap cooling [6, 7]. Thermophilic composting is an efficient, sustainable way of degrading and bio-converting raw organic waste into a stabilized, humic substance [8, 9]. Furthermore, this composting method is a reservoir of novel bacteria and thermostable enzymes useful in soil nutrient cycling and industrial applications [6, 10]. Studying the vast community and functional diversities of compost microbes is necessary to understand their specific colonization capacity/community structure during the utilization of diverse, complex materials such as dry maize stalks, *Lantana camara*, and *Tithonia diversifolia*. Input materials (feedstock) are vital in the composting process since they directly influence microbial diversity and contribute to biodegradative processes and, ultimately, the final product of the composting process. Compared to a single material type, combining composting materials such as cattle manure, maize stalk, and green material (e.g., *Lantana* and *Tithonia*) is recommended to ensure a nutrient-rich product [11, 12]. *Lantana camara* and *Tithonia diversifolia* have been used to enhance the levels of Nitrogen, Phosphorous, and Potassium in manure during composting [13]. These leguminous plants are readily available and have been adopted by farmers for green manure and compost production [14, 15]. These materials have, however, been reported to inhibit microbial proliferation, with studies focusing on their growth habitats and the pathogenic microbes, in vitro [16–18]. Therefore, there is a need to investigate these materials’ individual and collective influence on a dynamic ecosystem such as the composting environment.

The composting period also directly influences shifts of beneficial and pathogenic microbes within composting environment. Mature compost has been documented to have a higher abundance of beneficial microbes and less pathogenic groups such as *Staphylococcus*, *Klebsiella*, *Enterobacter,* and *Serratia* [19–22]. However, the maturity days and compost quality are influenced by the nature of composting feedstock, with more complex material requiring more time and microbial categories and pathways to mineralize into the humic substance [3, 18].

Metagenomics, a culture-independent technique, gives information on community composition, diversity, metabolic potential, and functional profiles using genomic DNA extracted from environmental samples. Metagenomics allows ecologists to directly sequence DNA from the environment, providing insights into the community and functional profiles of microbes in-situ [23]. Metagenomics can be classified into whole-genome shotgun sequencing or marker gene, also referred to as targeted sequencing. The whole-genome shotgun sequencing approach provides insights into the genomic diversity, gene content, and functional potential of the microorganisms in each sample. On the other hand, the marker gene approach is based on sequencing a specific gene region. This approach allows the identification of microbial composition based on the taxonomic groups present in the sample [24]. Despite the superior insights that whole-genome shotgun sequencing provides, much higher costs are associated with this approach than the marker gene approach. Bioinformatics tools such as Phylogenetic Investigation of Communities by Reconstruction of Unobserved States version 2 (PICRUSt 2) have been developed and customized to rely on marker gene sequence outputs from pipelines such as Denoising Algorithm version 2 (DADA2) to predict environmental microbial populations’ genomes and functional capabilities [25]. DADA 2 taxonomically classifies DNA sequences based on resultant amplicon sequence variants (ASVs). ASVs have higher resolution than traditional operational taxonomic units (OTUs) [26]. ASVs use a denoising approach to cluster organisms based on sequence probability rather than sequence identity, as has been the case with OTU clustering [27].

This study used Illumina sequencing of 16S rRNA genes to investigate prokaryotic communities, syntrophic associations, and metabolic functions under four compost regimes. This research hypothesized that different compost regimes and composting periods had diverse influences on prokaryotic community structure and function. This study sought to establish the prokaryotic community structure, functional profiles, and microbial interactions influenced by various green composting regimens and periods using amplicon sequencing.

## Methods

### Compost heaping and sampling

Compost preparation and heaping were done at Farming Systems Comparison in the Tropics; Kenya (SysCom Kenya) site at Thika, Kenya (01° 0.231′ S 37° 04.747′ E) within the ongoing long-term farming systems comparison trials (www.system-comparison.fibl.org) [28, 29]. The trial was established by the Research Institute of Organic Agriculture (FiBL) and local partners, International Centre for Insect Physiology and Ecology (icipe) and Kenyan Agricultural and Livestock Research Organization (KALRO) to compare productivity, profitability, and sustainability of organic and conventional farming systems in the tropics (www.system-comparison.fibl.org, [29]).

The design shown in Table 1 was adopted for treatments, which were informed by common farmer practices in Kenya and the availability of nitrogenous material for composting in the region [28, 30].

**Table 1:** Compost preparation, experimental treatments, and ratios of associated feedstock

Source of variation	Feedstock	Ratio	Treatment/sample description
Lantana (L)	Fresh cow dung manure, dry maize stalks, Lantana twigs	(4:2:1 w/w)	L1- Lantana-based compost at 21 days of composting L2- Lantana-based compost at 42 days of composting L3- Lantana-based compost at 63 days of composting L4- Lantana-based compost at 84 days of composting
Tithonia (T)	Fresh cow dung manure, dry maize stalks, Tithonia twigs	(4:2:1 w/w)	T1- Tithonia-based compost at 21 days of composting T2- Tithonia-based compost at 42 days of composting T3- Tithonia-based compost at 63 days of composting T4- Tithonia-based compost at 84 days of composting
Grass (G)	Fresh cow dung manure, dry maize stalks, Grass clippings	(4:2:1 w/w)	G1- Grass-based compost at 21 days of composting G2- Grass-based compost at 42 days of composting G3- Grass-based compost at 63 days of composting G4- Grass-based compost at 84 days of composting
Lantana + Grass + Tithonia (LTG)	Fresh cow dung manure, dry maize stalks, Lantana twigs, tithonia twigs, and Grass clippings (in the ratio of 1:1:1)	(4:2:1 w/w)	LTG1- Lantana + Grass + Tithonia-based compost at 21 days of composting LTG2- Lantana + Grass + Tithonia-based compost at 42 days of composting LTG3- Lantana + Grass + Tithonia-based compost at 63 days of composting LTG4- Lantana + Grass + Tithonia-based compost at 84 days of composting

Wood ash (1 kg) and soil (5 kg) were sprinkled after every layer was heaped

Composting materials such as fresh cow dung manure, dry maize stalks, wood ash, and soil were common to all compost heaps, thus sourced from the same facility to avoid differences in the initial composition of feedstock material. Fresh cow dung manure was sourced from a dairy unit near the LTE Thika site, while dry maize stalks were from the preceding season of the Long-term experiment at Thika. Maize stalks, tithonia, grass, and lantana twigs were individually cut into small pieces (3-5 cm long) to enable uniform and faster breakdown. The experiment was conducted between September 2020 and December 2020.

Triplicate compost heaps of each treatment were prepared on a flat, sheltered composting surface that was devoid of surface runoff. Compost heaping was done by layering material where; small dry twigs were laid on a flat leveled composting surface, followed by dry chopped maize stalks, cow dung manure from zero-grazed cattle, and finally green material (Lantana/Tithonia/Grass/ mixture of Lantana, Tithonia, Grass). The heap moisture content was adjusted to about 60%, as recommended at the beginning of composting [31]. Compost heaps aeration was done by turning every 4 days during the first 20 days: and weekly for the following days till 84 days of composting.

### Analysis of physical-chemical characteristics

The temperature was monitored daily using a compost thermometer (model: WIKA 110824862-EN 13190). This data was collected from three locations on the heap by inserting the thermometer halfway between the top and bottom of the pile to the maximum probe depth (45 cm). Compost samples for other physical-chemical parameters analysis were collected every 21 days till the entire composting period of 84 days. The 21st composting day was used as the baseline since composting materials (maize stovers, cow dung manure, and lantana/grass/tithonia) were not sufficiently homogenized during the earlier days of composting. Therefore, compost samples were collected at 21, 42, 63, and 84 days of composting. The pH of the compost (1:10 w/v waste: water extract), moisture, germination index, and carbon dioxide emission during sampling days (mg CO_2_ g-1d-1) were done as described by Adamtey [32]. Total Kjeldahl Nitrogen (TKN) was analyzed using the Kjeldahl method. The total organic carbon and total phosphorus (TP) (Olsen P) were analyzed according to Okalebo [33].

### Sampling for 16S rRNA analysis of compost prokaryotic communities

Compost samples for 16S rRNA extraction were collected simultaneously for the physical-chemical analysis described above (at 21, 42, 63, and 84 days of composting). Samples were collected from five different positions of each compost heap using a sharp shovel that was, pre-cleaned with 70% ethanol, making pre-measured cuts into the windrow pile from the peak of the pile down to the bottom [34]. The samples were put into sterile 200 g containers and transported to SysCom Kenya’s sample room at icipe for storage at − 20 °C before total DNA extraction at the Kenyatta university Plant transformation laboratories, Kenyatta University.

### DNA extraction and amplification

Total compost DNA was extracted from triplicate compost subsamples per treatment replicate. Extraction was done using the PureLink™ Microbiome DNA Purification Kit (Catalog number: A29790) as per the manufacturer’s instructions. DNA quality and concentration per sample were confirmed using NanoDrop (Maestrogen) and visually under 2% agarose gel. The extracted compost DNA was shipped under dry ice to the Molecular Research DNA Lab (www.mrdnalab.com, Shallowater, TX, USA) for downstream processing.

The 16S rRNA gene V4 variable region PCR primers 515F (5′-GTGCCAGCMGCCGCGGTAA-3′) and 806R (GGACTACNVGGGTWTCTAAT) were used in PCR using the HotStarTaq Plus Master Mix Kit (Qiagen, USA). The following conditions were used for 16S rRNA gene amplification: Initial denaturation heating at 95 °C for 5 min, followed by 30 cycles of denaturation at 95 °C for 30 s, annealing at 53 °C for 40 s, and extension at 72 °C for 1 min, and final elongation at 72 °C for 10 min. After amplification, PCR products were checked in 2% agarose gel to determine the amplification success and the bands’ relative intensity. Equimolar quantities of PCR amplicons obtained from 16 individual composts were multiplexed using unique indices, pooled, and sequenced using Illumina MiSeq next-generation technology at MR DNA (www.mrdnalab.com, Shallowater, TX, USA).

### Statistical analysis of physical-chemical parameters

The resulting measurements of physical-chemical analysis were individually subjected to a normality test using the Shapiro test before means separation using ANOVA under the agricolae package (version 1.3–5). Comparing the compost treatments was done per composting day for each physical-chemical parameter, followed by a Tukey posthoc.

### Bioinformatics and statistical analysis of 16S rRNA sequence data

After sequencing, barcodes and amplicon primer sequences were trimmed, after which low-quality sequences were denoised and filtered out. Reads with < 200 base pairs after phred20-based quality trimming, sequences with ambiguous base calls, and those with homopolymer runs exceeding 5 bp were removed [35].

The raw 16S rRNA sequences were submitted to the NCBI sequence read archive with accession number PRJNA822850; (https://www.ncbi.nlm.nih.gov/sra/PRJNA822850). The sequence data were analyzed in R (version 4.1), Divisive Amplicon Denoising Algorithm 2 (DADA2) version 1.20.0 workflow [27]. The “filterAndTrim” function in DADA2 was used to filter the reads [36], after which error rates for each sample were computed. Subsequently, the “derepFastq” function was used to dereplicate the reads inferring associated amplicon sequence variants (ASVs) and their abundance. Spurious sequence variants were eliminated by merging forward and reverse reads and removing chimeric ASVs using the “mergePairs” and “removeChimeraDenovo” functions [27]. Finally, the “assignTaxonomy” function utilized the SILVA (version 138) reference database obtained from zenodo to assign ASVs to corresponding taxonomies. ASVs are amplicons resulting from the removal of amplification and sequencing errors. They distinguish the variation of sequences by a single nucleotide change [27, 36]. Further, classification was done using the Refseq-ribosomal database project (RDP) [37]. The Refseq-RDP classifications were combined with those from SILVA 138 using the “cbind” function. Multiple sequence alignment was done on ASV sequences not classified to genus level using the “AlignSeqs” function [38]. The taxonomy table was merged with the abundance table. Taxa prevalence was done based on the absolute abundance data to determine the predominant taxa present in the compost samples. The taxa prevalence was plotted by taxonomic subsetting of phyla counts using the “subset” function followed by plotting using “ggplot” function in the ggplot2 package. The relative abundance and bar plots of the top 20 most abundant prokaryotic classes were plotted using the phyloseq package (version 1.36.0). A table of all prokaryotic genera in the compost samples was also output using the “write.table” function.

Alpha diversity estimate was done from a rarefied phyloseq object, using “Observed” “Shannon”, and “Simpson” metrics to determine the diversity and richness of the four compost treatments and sampling days. The Shapiro and significance tests were calculated before plotting the alpha diversity metrics. Venn diagrams were used to estimate the beta diversity among composting treatments and days. Key environmental drivers of prokaryotic communities at the class level were also determined by computing the Canonical Correspondence Analysis (CCA) using the “plot” function of the vegan 2.6–2 package.

The absolute abundance data were used to construct an undirected co-expression network of compost microbes. Using Pearson correlation, this was done by establishing positive co-occurring microbes from the OTU count table and taxonomy file. Then, the Fruchterman Reingold layout algorithm was used for network layouts, such as node placement, before plotting using “plot_net” function in the ggplot2 package (version 3.3.5) [38].

Phylogenetic investigation of communities by reconstruction of unobserved states (PICRUSt2) (version 2.4.1) pipeline was used to predict functional abundances based on ASVs marker gene sequences [25]. The resulting Enzyme Commission (EC) classification abundances were used for statistical comparison of different compost treatments and days. Lastly, principal component analysis (PCA), heat maps, and boxplots of pathways responsible for composting were visualized using statistical analysis of taxonomic and functional profiles (STAMP) (version 2.1.3) software [39].

## Results

### The type of green feedstock influences the succession of compost physical-chemical properties

There were no significant differences (*P* > 0.05) in pH among compost treatments during the early stages of composting (21 days). Still, apparent differences were reported at the end of the composting period (84 days). Grass-based compost (G) had the highest pH (8.72) among the different compost types at the end of the composting period (Table 2).

**Table 2:** Physical-chemical characteristics of compost treatments on various composting days

Parameter	Sampling Time (day)	G	L	LTG	T	Significance
pH	21	8.41^a^	8.50^a^	8.48^a^	8.48^a^	**ns**
	42	8.95^ab^	9.00^a^	8.90^b^	8.99^a^	**
	63	8.37^a^	8.36^a^	8.25^a^	8.47^a^	**ns**
	84	8.72^a^	8.62^c^	8.55^d^	8.67^b^	***
Organic Carbon (%)	21	22.73^c^	28.00^a^	26.40^b^	25.77^b^	***
	42	17.67^a^	17.17^ab^	16.07^bc^	15.33^c^	**
	63	16.40^a^	15.57^ab^	15.20^ab^	14.47^b^	*
	84	13.75^a^	13.67^a^	12.87^c^	13.27^b^	***
Potassium (%)	21	1.42^b^	1.52^b^	1.58^ab^	1.78^a^	**
	42	1.26^a^	1.16^b^	1.19^ab^	1.21^ab^	*
	63	1.07^a^	1.10^a^	1.10^a^	1.09^a^	**ns**
	84	1.09^a^	1.04^b^	1.01^b^	1.09^a^	***
Total Nitrogen (%)	21	0.77^b^	0.96^a^	0.82^ab^	0.97^ab^	*
	42	0.75^a^	0.68^ab^	0.67^ab^	0.59^b^	*
	63	0.62^a^	0.58^a^	0.59^a^	0.49^a^	**ns**
	84	0.55^a^	0.60^a^	0.54^a^	0.55^a^	**ns**
Total Phosphorous (%)	21	0.18^b^	0.21^ab^	0.24^a^	0.22^ab^	*
	42	0.18^a^	0.14^b^	0.13^b^	0.14^b^	***
	63	0.16^a^	0.15^a^	0.15^a^	0.15^a^	**ns**
	84	0.17^a^	0.16^b^	0.14^c^	0.16^ab^	***
Temperature (°C)	21	41.3^a^	39.7^a^	38.3^a^	38.3^a^	ns
	42	24.3^ab^	23.0^b^	26.3^a^	25.0^ab^	**
	63	25.0^a^	25.0^a^	25.0^a^	27.0^a^	ns
	84	22.7^a^	22.7^a^	23.0^a^	22.3^a^	ns
Moisture Content (%)	21	72.2^a^	73.9^a^	70.9^a^	72.3^a^	ns
	42	46.7^b^	51.1^a^	44.9^c^	51.6^a^	***
	63	17.1^a^	18.8^a^	19.6^a^	18.8^a^	ns
	84	18.3^a^	19.3^a^	19.3^a^	19.8^a^	ns

Values with the same superscripts were not significantly different

*ns* not significant

*P ≤ 0.05; ** *P* ≤ 0.01 and *** *P* ≤ 0.001

Carbon levels decreased during the composting period. Differences in carbon levels in the composting treatments were observed from the initial to later stages. There were no differences (*P* > 0.05) among the various treatments regarding nitrogen levels at the end of the composting process. However, Lantana-based compost (L) recorded higher nitrogen levels (0.60%) compared to other treatments (Table 2).

Nitrogen levels were highest in Tithonia-based compost at 21 days of composting (0.97%). However, this was not significantly different (*P* > 0.05) compared to Lantana and mixed (LTG) composts. The regimen, however, recorded the least nitrogen levels at 42 days and the highest decline in nitrogen levels (0.97% at 21 days to 0.59% at 42 days), Table 2.

Temperature and moisture levels were not significantly different among the composting treatments at 21, 63, and 84 days. Significant differences were recorded for these two parameters at 42 days, with mixed compost (LTG) recording the highest temperature levels (26.3 °C). Tithonia-based compost had the highest moisture content at 42 days of composting (51.6%), which was not significantly different from that of Lantana-based compost (51.1%), Table 2.

### Different green composting sources exhibit distinct microbial biodiversity and community structures

After demultiplexing, quality filtering, denoising, and chimera removal, 4,198,194 high-quality reads were obtained from 5,957,427 16S rRNA reads (Supplementary Table 1) all the compost samples, which generated a total of 1813 ASVs. Ninety-nine percent (99%) of the resultant ASVs had a sequence length of about 250 bp (Supplementary Fig. 1). Out of these reads, grass (G), lantana (L), tithonia (T), and their combination (LTG) based compost accounted for 622,573, 802,660, 646,500, and 666,617 ASV copies, respectively (Supplementary Table 1). A total of 25 Phyla, 51 classes, 246 families, and 338 genera were recovered from the 16S rRNA amplicon sequence variants (Supplementary Table 2).

### Prokaryotic prevalence in the compost

**Fig. 1:**
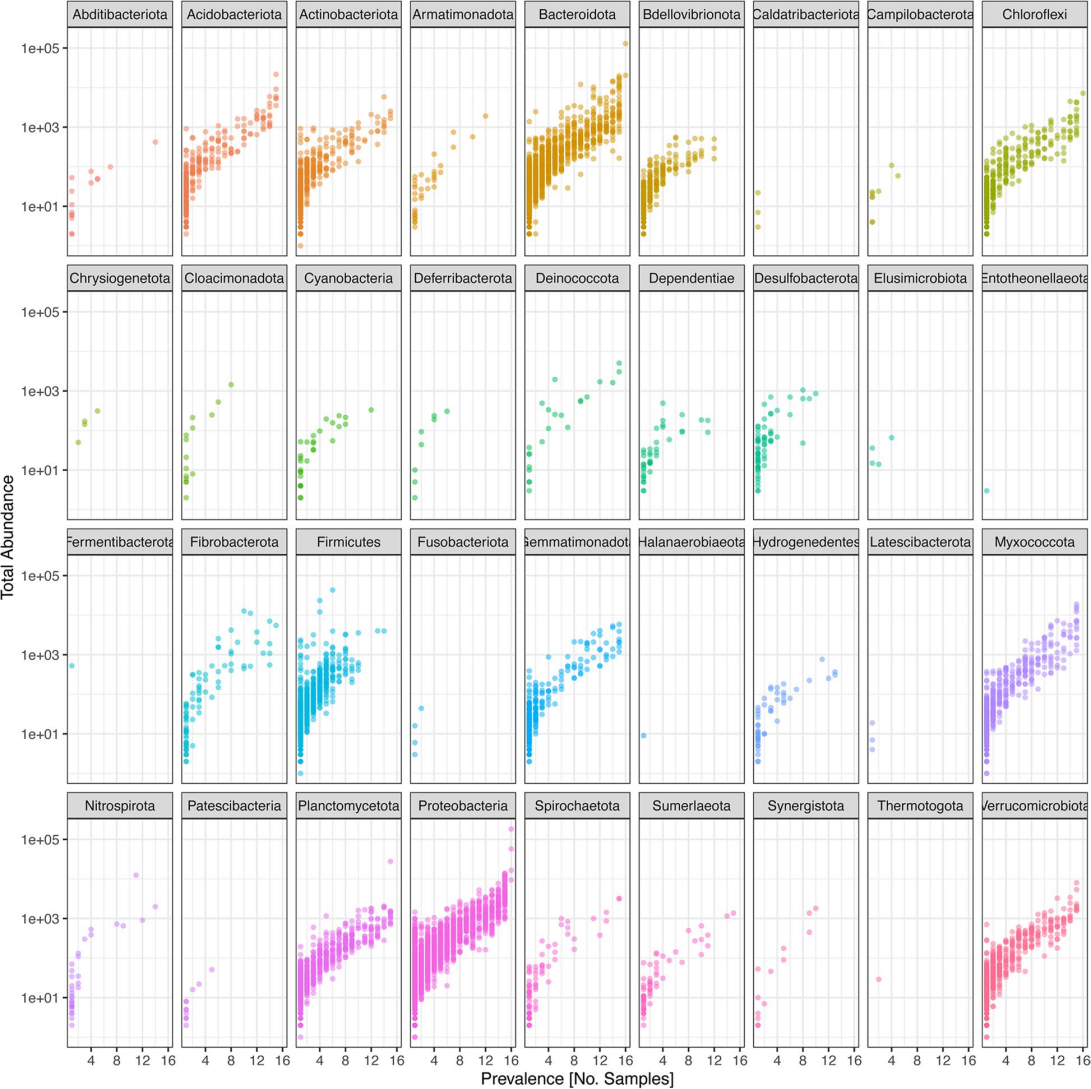
Feature prevalence of major taxa in different compost samples. This is an evaluation of the overall distribution of different prokaryotic taxa among the different compost treatments on different composting days. In total, we are evaluating prevalence in 4 compost treatments (lantana-based, tithonia-based, grass-based, and mixed compost (lantana + tithonia + grass; based) sampled at 4 composting periods (21, 42, 63, and 84 days)

Phylum-level classification of prokaryotic communities using the feature prevalence of the 16S datasets of the compost samples showed the overall distribution of prokaryotic phyla among various samples (Fig. 1). Each point on the plot corresponds to a different or unique taxon per prokaryotic phylum, within the number of samples (x-axis). In this study, *Entotheonellaeota*, *Halanaerobiaeota*, *Fermentibacterota*, and *Thermotogota* only appeared once in less than two compost samples. Moreover, the predominant phyla included *Acidobacteriota*, *Actinobacteriota*, *Bacteroidota*, *Chloroflexi*, *Myxococcota*, *Planctomycetota*, *Proteobacteria*, and *Verrucomicrobiota*. These were present in all compost treatments at all stages of composting (Fig. 1).

### Taxonomic composition of prokaryotic communities

**Fig. 2:**
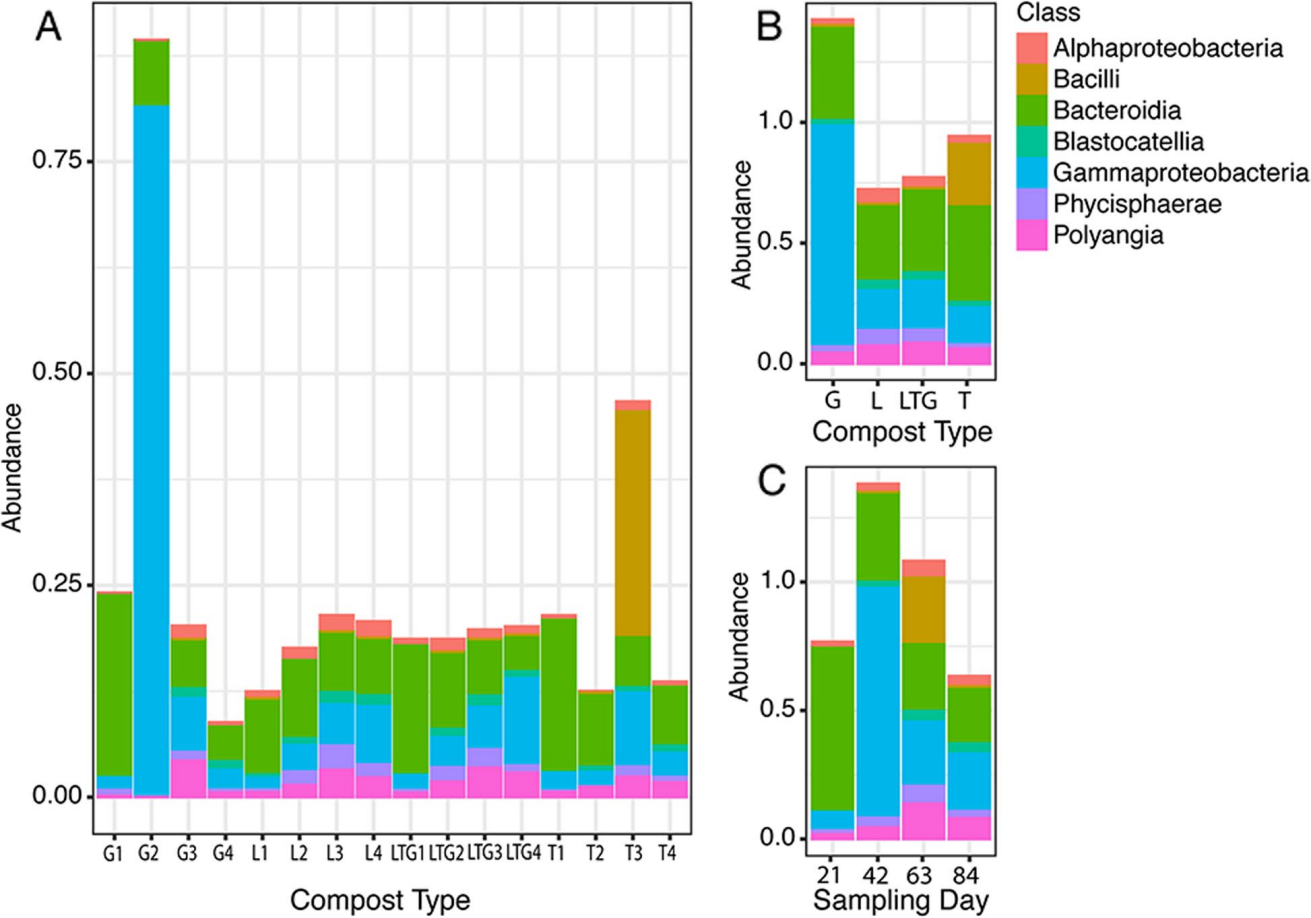
**A** Relative abundance of the most abundant prokaryotic classes as influenced by compost treatments and days; G is Grass-based compost, L is Lantana-based compost, LTG is Lantana + Tithonia + Grass based compost, T is Tithonia-based compost; G1, G2, G3, G4 is Grass-based compost at 21, 42, 63 and 84 days of composting, L1, L2, L3, L4 is Lantana-based compost at 21, 42, 63 and 84 days of composting, LTG1, LTG2, LTG3 and LTG4 is Mixed compost (Lantana + Tithonia + Grass) based compost at 21, 42, 63 and 84 days of composting, T1, T2, T3, T4 is Tithonia-based compost at 21, 42, 63 and 84 days of composting; **B** Effect of different compost treatments on the relative abundance of top prokaryotic classes; **C** Combined effect of composting treatments on the relative abundance prokaryotic classes at 21, 42, 63 and 84 days of composting

*Bacteroidia* was the most abundant and ubiquitous prokaryotic class in all treatments and composting days except in grass-based compost at 42 days of composting (G2). It was more abundant in most treatments on the 21st day of composting than on other composting days. During the 21st day of composting, Grass-based compost (G1) had the highest relative abundance of this class, and Lantana-based compost (L1) had the least relative abundance of this class among all the treatments (Fig. 2A).

Grass-based compost recorded the highest overall abundance of Prokaryotic classes, with comparably higher levels of *Gammaproteobacteria* and *Bacteroidia*. On the other hand, Lantana-based compost had the least overall abundance of prokaryotic classes (Fig. 2B). On the other hand, 42 days of composting had the highest relative abundance of prokaryotic classes compared to other composting days (Fig. 2C).

We observed generally low abundances of taxa known to include pathogenic bacteria across all the compost treatments along the composting period. Such recorded bacteria include *Treponema*, a known human pathogen (Supplementary Table 3).

### Alpha and beta diversity metrics by compost type and composting day

**Fig. 3:**
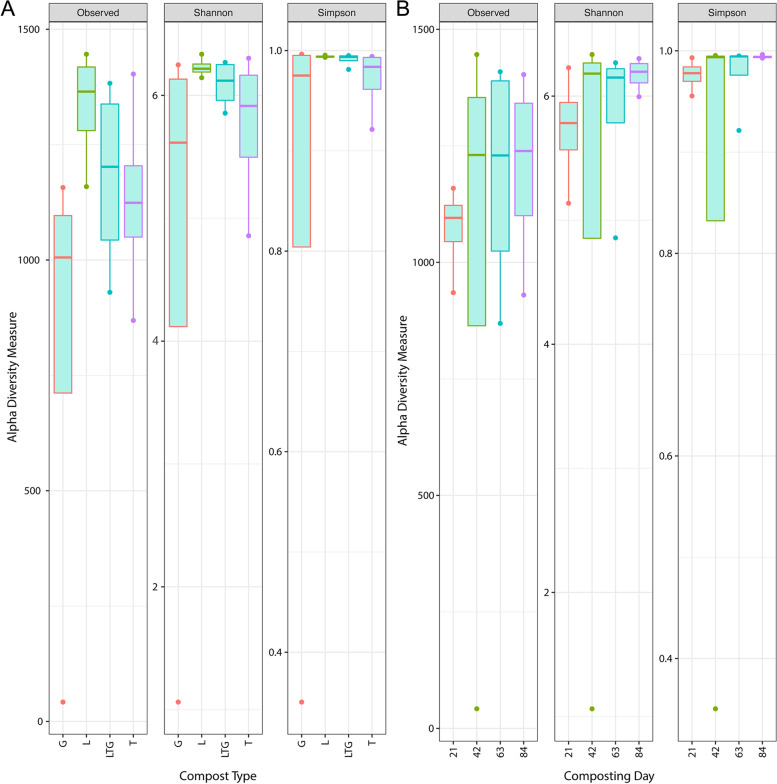
Alpha diversity (Observed, Shannon and Simpson) of prokaryotic communities under different composting treatments (**A**) and composting days (**B**). L, T, G, and LTG represent Lantana, Tithonia, Grass and mixed (Lantana + Tithonia + Grass) based composts, respectively. Day 21, day 42, day 63, and 84 represent the effect of combined compost treatments at 21, 42, 63, and 84 days

The alpha diversity index showed a significant impact of compost treatment on prokaryotic populations (Fig. 3A). Minimal variation was observed in L- and LTG-based composts (Fig. 3A). Composting days influenced alpha diversity, with the most significant variability observed on the 42nd day of composting (Observed, Shannon and Simpson). The least variability was observed at 21 days of composting (Observed). However, Shannon and Simpson diversity indices showed the least variability at 84 days of composting (Fig. 3B).

**Fig. 4:**
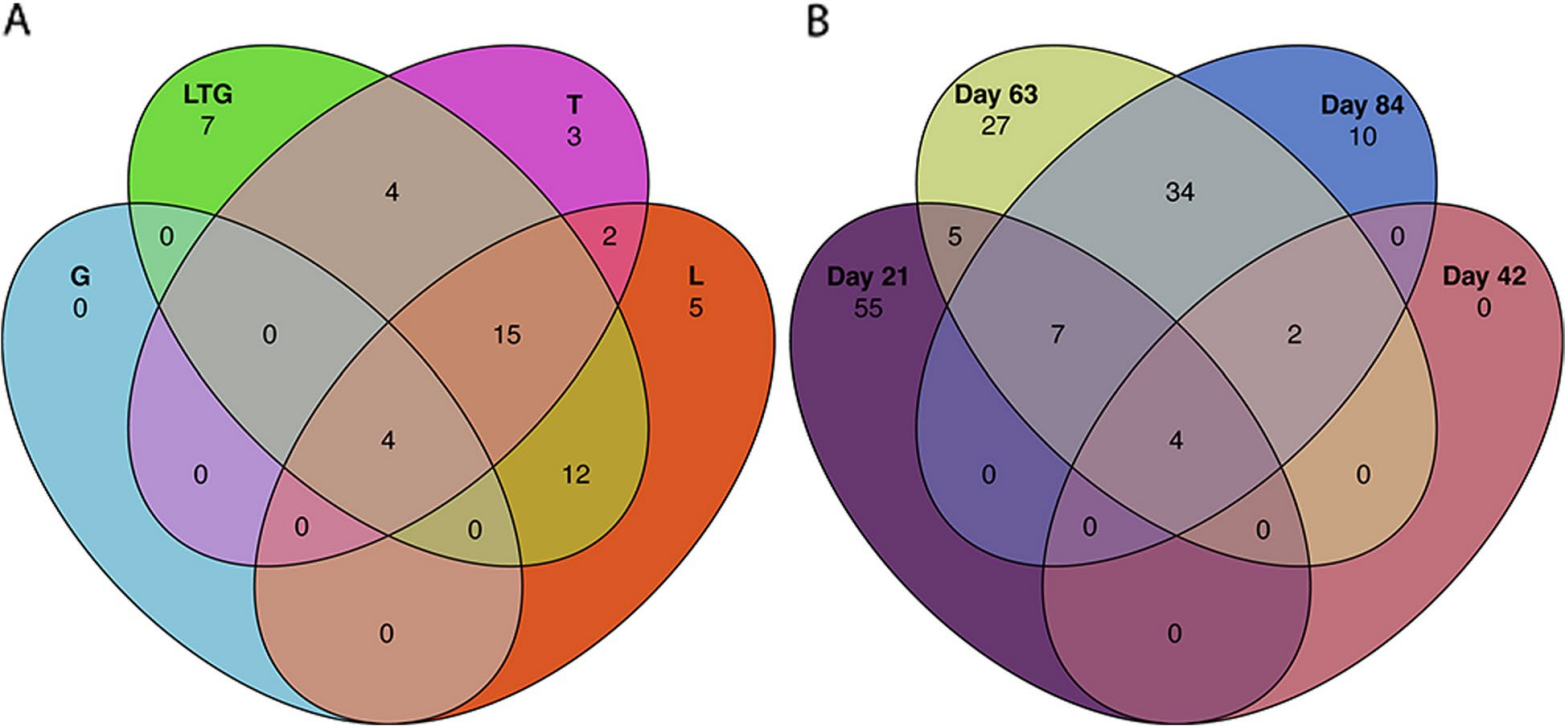
Venn diagrams showing the distribution of unique and shared ASVs within various composting treatments (**A**), and composting days (**B**). The number of ASVs in each compost type and the composting day are indicated in the respective circle. L, T, G, and LTG represent lantana, tithonia, grass and mixed (lantana + tithonia + grass) base compost respectively. Day 21, day 42, day 63, and day 84 represent the effect of combined compost treatments at 21, 42, 63, and 84 days

Four prokaryotes were common to all compost regimens and composting days (*Acidibacter* spp., *Hydrogenophaga temperata*, *Ruminofilibacter xylanolyticum*, and a prokaryote from the *Saprospiraceae* family) (Fig. 4A and B). Grass-based compost (G) did not harbor any unique genera. A combination of Lantana, Grass, and Tithonia (LTG) based compost (L) recorded the highest unique genera (7). At the same time, L and T had 5 and 3 unique genera, respectively (Fig. 4A). The highest unique ASVs were recorded at 21 days of composting (55), while there were no unique ASVs at 42 days of composting (Fig. 4B). There were no shared ASVs between 42 days 21 days as well as 63 days.

### Physical-chemical properties drive the community structure of compost microbiomes

**Fig. 5:**
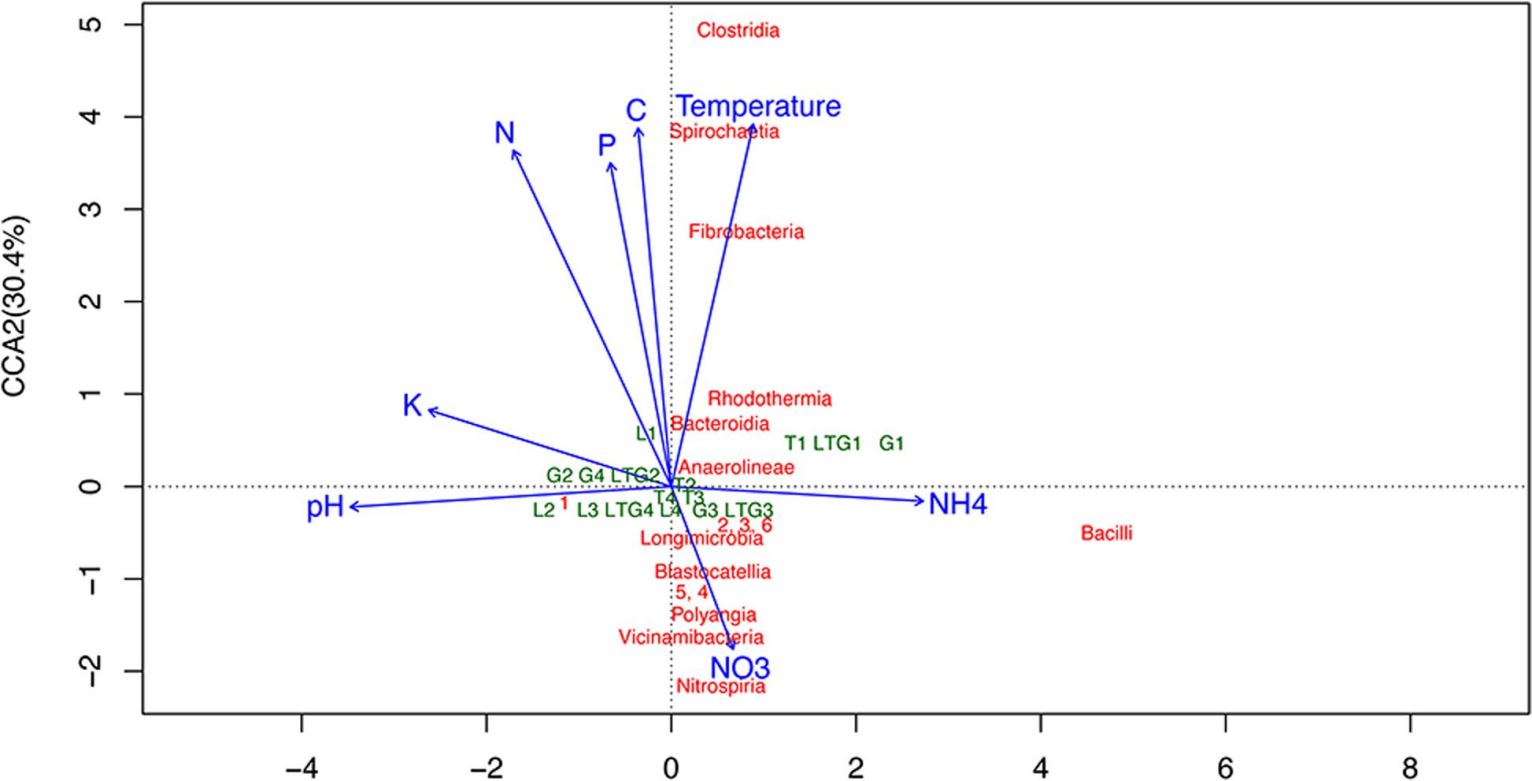
Canonical correspondence analysis (CCA) of physical-chemical characteristics and top 20 prokaryotic classes in different compost samples. *K – Potassium, C – Carbon, NO3 – Nitrates, NH4 – Ammonia, and P – Phosphorous; 1 – Gammaproteobacteria, 2 – Alphaproteobacteria, 3 – Verrucomicrobiae, 4 – Phycisphaerae, 5 – Gemmatimonadetes, 6 – Planctomycetes, and 7 - Actinobacteria

Analysis of physical-chemical variables in combination with prokaryotic diversities among the samples resulted in an ordination plot. As shown in Fig. 5, the first two canonical axes explained 44 and 32% of the variation. Class *Bacilli* was the most sensitive taxa to ammonia, while class *Nitrospiria* was the most sensitive to nitrates. Nitrate and pH influenced the grouping of most compost samples closer to each other. N, P, C and K had little influence on the grouping of prokaryotes in the various compost samples (Fig. 5).

### *Bacteroidia* is the most represented prokaryotic class in compost microbial association networks

**Fig. 6:**
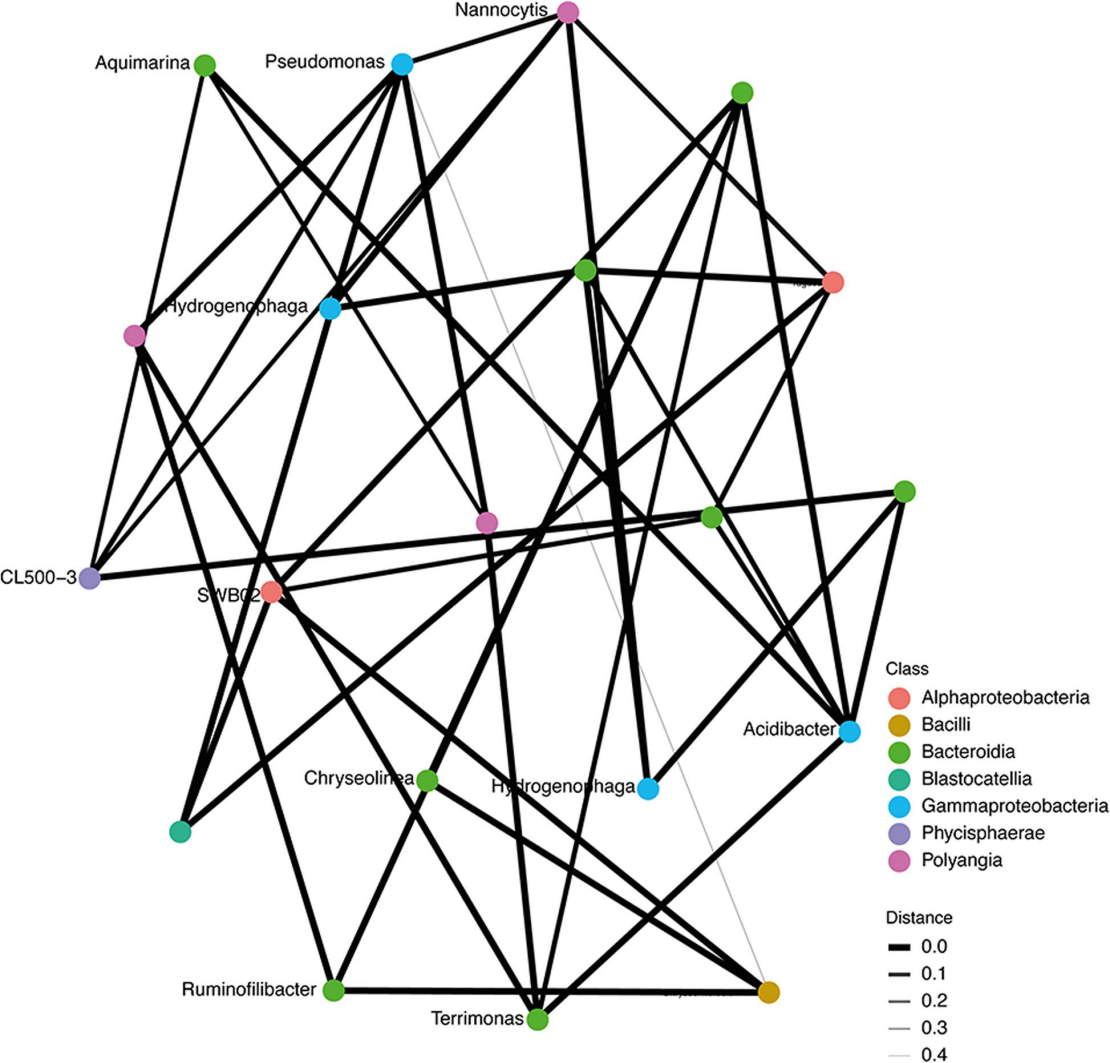
A correlation network showing interactions among prokaryotic classes driving composting. The network nodes represent genera whereas the edges represent microbe-microbe interaction weights. Various color codes in the networks represent labeled genera that are within the classes listed on grid

The relationship between various compost bacterial microbiota is presented in an interaction network based on the correlation of the most abundant bacterial microbiota during compost degradation and maturation (Fig. 6). The thick edges (with a distance of 0.0) represent a close interaction between the taxa. The nodes in green and blue colors represent highly ubiquitous and co-occurring prokaryotes of *Bacteroidia* and *Gammaproteobacteria* taxa at the class level. The edges with boldness ranging from 0.0 to 0.5 represent association co-dependence of different classes, where 0.0 and 0.5 show high and insignificant co-dependence, respectively. Among bacterial communities, *Pseudomonas* of class *Gammaproteobacteria* had the most interactions with other prokaryotic genera. However, it had the least correlation with *Chrysomicrobium* of the class *Bacilli* (Fig. 6). In the prokaryotic interaction network, the class *Alphaproteobacteria* had two (2) genera (SWB02 and *Tagaea*), and *Bacilli* had one genus (*Chryseomicrobium*) that had major roles in community networks. On the other hand, class *Bacteroidia* had six (6) genera (*Aquimarina*, *Chryseolinea*, *Ruminofilibacter*, *Terrimonas*, and an *Unknown* genus), while *Blastocatellia* had one genus (*Blastocatellaceae*), *Gammaproteobacteria* class had three (3) genera (*Acidibacter*, *Hydrogenophaga*, and *Pseudomonas*) involved in major networks in the compost environment; Fig. 6.

### Different green feedstock material influence compost functional pathways similarly

**Fig. 7:**
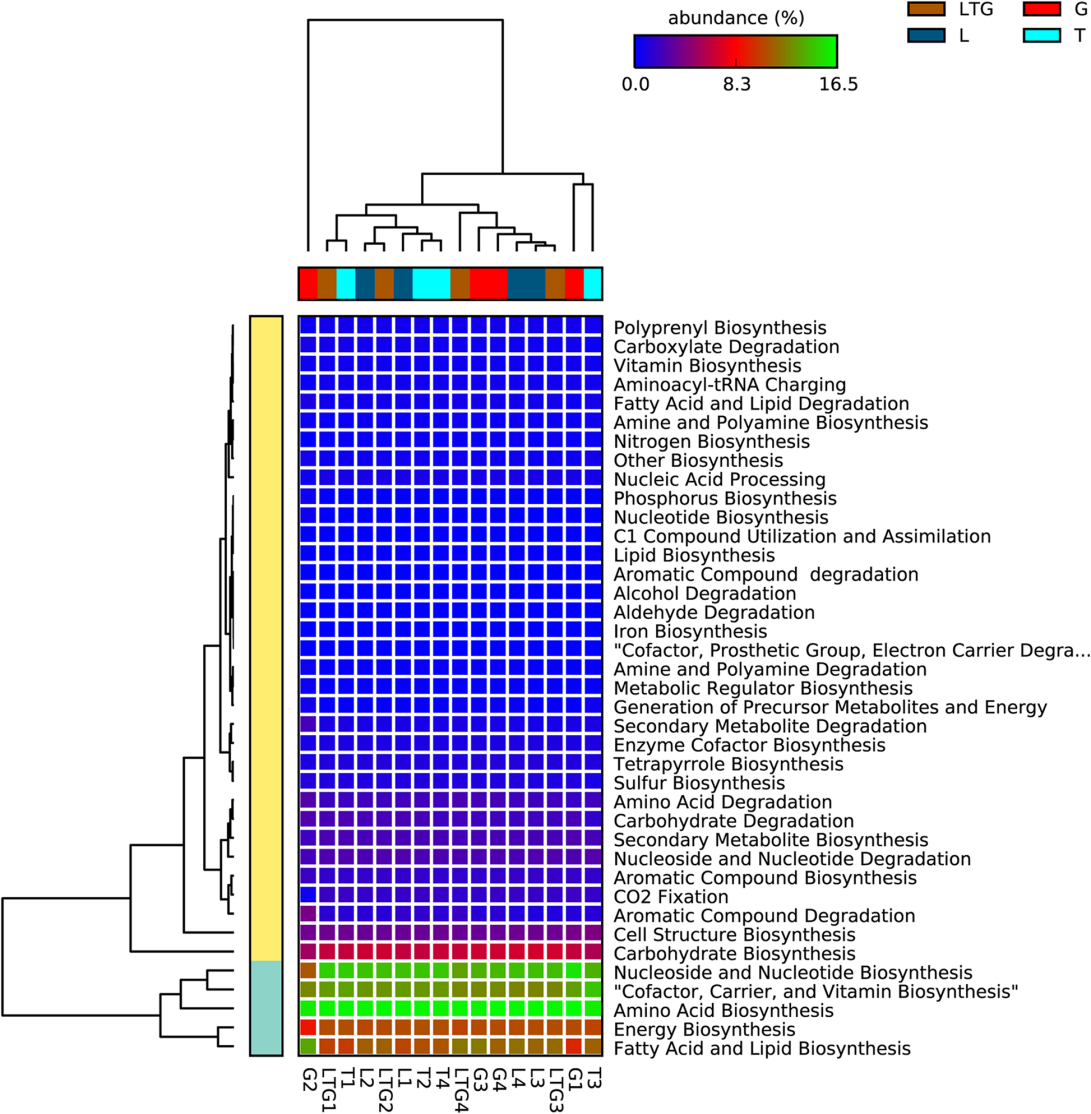
Heatmap of the normalized relative abundances of the predicted functional categories of microbiomes associated with various composting treatments and days. Functional categories were predicted using PICRUSt2

Prediction of sequences associated with major functional metabolic pathways revealed the five most abundant pathways: nucleoside and nucleotide biosynthesis, cofactor, carrier and vitamin biosynthesis, amino acid biosynthesis, energy biosynthesis, and fatty acid and lipid biosynthesis. Carbohydrate biosynthesis and cell structure biosynthesis also ranked high in terms of abundance. Based on this hierarchical clustering, the various compost types and days were clustered into two main clades. G2 (Grass-based compost at 42 days) clustered alone, while LTG1, T1, L2, LTG2, L1, T2, and T4 clustered on the second clade. LTG4, G3, G4, L3, L4, and LTG3 clustered together. T3 and G1 clustered on one unique clade (Fig. 7).

**Fig. 8:**
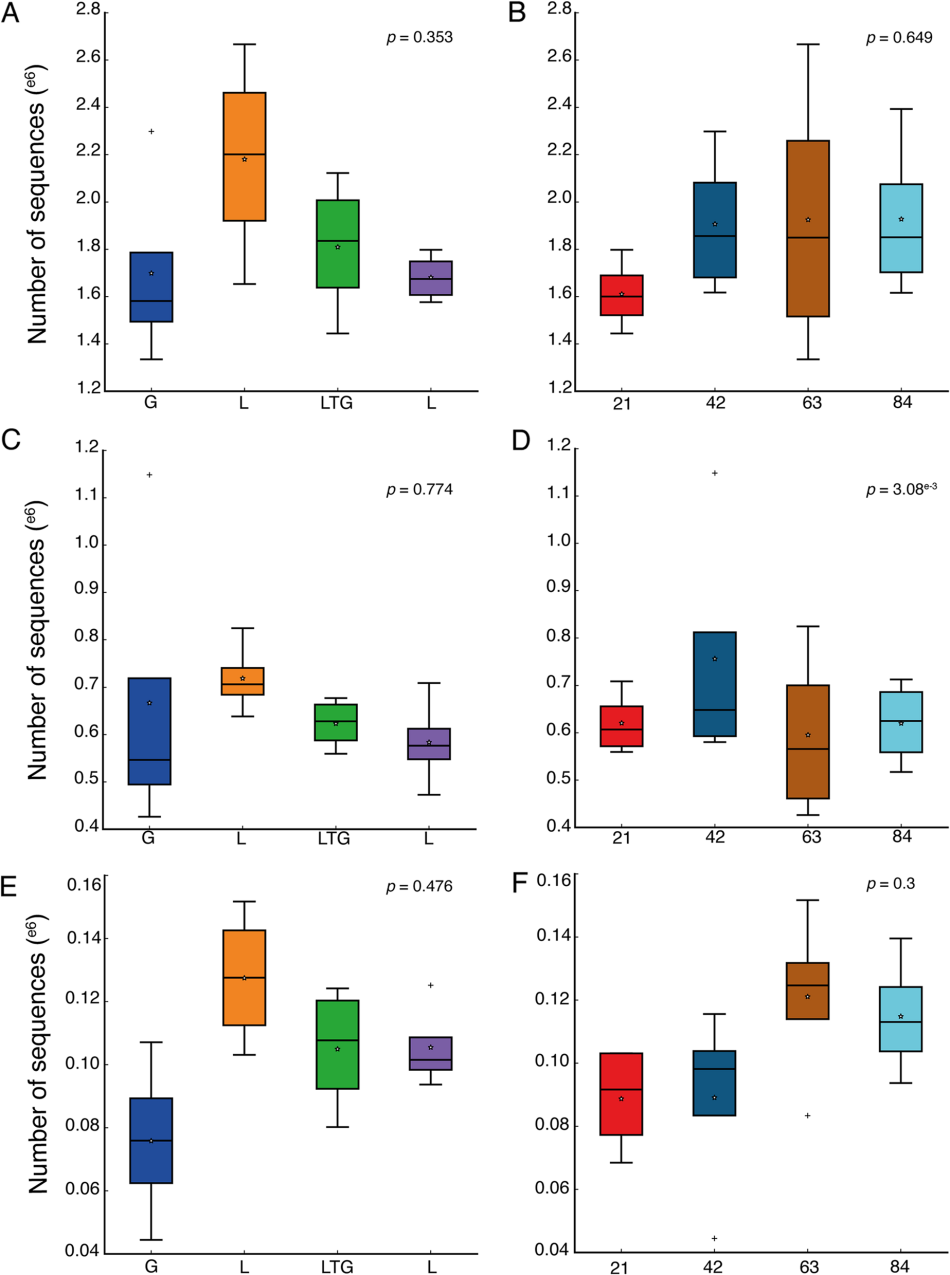
The abundance of prokaryotic sequences responsible for; carbohydrate biosynthesis as influenced by composting material (**A**) and composting days (**B**); carbohydrate degradation as influenced by composting material (**C**) and composting days (**D**); Nitrogen biosynthesis as influenced by composting material (**E**) and composting days (**F**)

This study revealed no significant (*P* = 0.353) difference among the treatments on carbohydrate biosynthesis; Fig. 8A. There was also no significant difference (*P* = 0.649) in carbohydrate biosynthesis among the composting days; Fig. 8B.

There were no significant differences observed among compost treatments concerning carbohydrate degradation. However, Lantana-based compost recorded the highest number of sequences responsible for carbohydrate degradation (Fig. 8C). There were significant differences among the composting days, with the highest carbohydrate degradative sequences recorded on the 42nd day of composting (Fig. 8D).

Despite the notable differences and shifts in Nitrogen levels recorded among the compost treatments and composting days, there was no significant difference in the abundance of sequences responsible for nitrogen biosynthesis (Fig. 8E, F).

## Discussion

This study focused on evaluating nutrient enrichment, prokaryotic diversity, and predicted metabolic succession during composting of cow dung manure as influenced by the source of green composting feedstock material and composting period.

Composting has been associated with nitrogen losses due to the related high temperatures despite the characteristic high nitrogen content in composting materials such as Tithonia and Lantana [40]. The high loss of total nitrogen in the compost environment is attributable to the high pile temperatures which is compounded by the fact that important nitrogen biosynthetic bacteria such as *Azotobacter chrococcum* cannot survive the high temperature that prevails [41]. The presence of these bacteria in the ecosystem could be attributed to nitrogen is fixation, reducing biogeochemical losses [42].

The dominance of *Acidobacteriota*, *Actinobacteriota*, *Bacteroidota*, *Chloroflexi*, *Myxococcota*, *Planctomycetota*, *Proteobacteria*, and *Verrucomicrobiota* has been reported in other studies [43]. These phyla are also regarded as resident in compost and are also attributed to the nature of composting material. These findings were consistent with other studies considering bacteria categories as residents across the composting period [44, 45]. These ubiquitous phyla have been reported to have an essential role in nutrient dynamics in the ecosystem [46]. The low dominance of *Entotheonellaeota*, *Halanaerobiaeota*, *Fermentibacterota*, and *Thermotogota* has also been reported in other studies [47].

ASVs determine environmental variations by classifying species groups based on their DNA sequence differences [36]. The highest number of ASVs observed in Lantana-based compost indicated the presence of diverse microbial communities attributable to the complexity of Lantana requiring more categories of prokaryotes to degrade compared to other composting treatments [40, 48].

The study enumerated the presence of class *Bacteroidia* which has species such as *Ruminofillibacter*, at all composting stages. *Bacteroiodia* species contribute to composting by degrading macromolecules such as cellulose, agar, and chitin [49]. The resurgence of *Acidibacter* at the later stages of composting affirms that sufficient composting enhances agriculturally essential microbes. Taxa such as *Acidibacter* have been reported to contribute to the biocontrol of plant diseases, plant growth, and modulation of plant response to abiotic stress [50]. The buildup of this genus towards the end of the composting process guarantees matures compost as a source of agriculturally beneficial microbes for application to the soils. The presence of an unknown genus in all our compost treatments points to composting being a source of agriculturally beneficial novel microbes [12]. The high abundance of taxa in sample T3 can be attributed to the relatively low Carbon and Nitrogen levels in this treatment compared to other samples. The comparatively low levels of these nutrients point to utilization by microbes [51].

The general decline in pathogenic prokaryotes, such as *Staphylococcus* and *Treponema*, confirms that extended composting periods are necessary to sanitize the compost of pathogenic microbial communities. Babajide et al. [14] reported that these categories were present during the first month of composting, with very minimal levels of *Treponema*.

The least intra-treatment variability in Lantana-based Compost (L) is attributable to the inhibitory influence of *Lantana camara* on microbial growth and diversity [52, 53], which did not allow meaningful changes in microbial diversity along the composting period. The relatively low variability in the mixed Compost (LTG) is also attributable to the *Lantana camara*. The application of *Lantana camara* during composting has been attributed to the reduction of prokaryotic diversity [29].

This study uncovered unique ASVs present in Lantana, LTG, and T-based compost, indicating the complexity of these materials compared to Grass [54–56]. The unique ASVs in mixed regimen compost (LTG) is attributable to the contribution of Lantana and Tithonia material in the mixed compost. It was observed that 42 days had no unique ASVs compared to other composting days. Therefore, this depicts this composting time as the decomposition process’s transition phase.

The high relative abundance of *Pseudomonas* in grass-based compost after 42 days (G2) shows that at this time, the compost harbored this genus which has been regarded as one of the free-living microbial genera that produce metabolites, such as siderophores and antibiotics, with specific suppressive activity against soil-borne pathogens [57]. However, positive shifts in the relative abundance of this genera were observed in the remaining compost treatments after 63 days of composting. This implies that the composting period positively influenced the abundance of this genus. The findings of this study showed that the class *Bacteroidota* was persistent along the composting period across all compost treatments. Previous studies have reported similar trends regarding this taxon [58].

The syntrophic interaction between prokaryotes in the compost environment plays a critical role in nutrient cycling, microbial buildup, and persistence in the ecosystem. *Gammaproteobacteria* and *Bacteroidia* are the integral classes driving cattle manure and green material co-composting. *Pseudomonas species* have been shown to have the highest interactions with most prokaryotic species. Studies have shown that *Pseudomonas* species break down complex carbohydrate material such as lignin into simpler biomolecules and substrates utilized by other prokaryotes in the degradation process [48, 59]. The study deduced that *Acidibacter species*, *Hydrogenophaga spp*, and *Pseudomonas* spp. of *Gammaproteobacteria* class; *Aquimarina spp*, *Chryseolinea* spp., *Ruminofilibacter spp*, and *Terrimonas* spp. of *Bacteroidia* class are the major drivers for biodegradation and maturation of compost.

Nucleoside and Nucleotide biosynthesis, cofactor, carrier and vitamin biosynthesis, Amino acid biosynthesis, and Fatty acid and lipid biosynthesis were shown as critical pathways responsible for microbial proliferation and buildup in compost. The Nucleosides and Nucleotides are required for cell growth and replication pathways, while amino acids, fatty acids, and lipids are building blocks for microbial cell structure [60–62]. Cofactors, carriers, and vitamins play a critical role in microbial enzymatic functions, ultimately directly influencing nutrient cycling through catabolic and anabolic processes within the available pool of nutrients in the compost environment [63–65]. The high abundance of energy metabolism pathways explains the rise in compost temperatures, generally higher than ambient temperatures until the compost is mature. Energy metabolism is a critical pathway in central carbon metabolism, hydrogen oxidation, and Nitrogen cycling through pathways such as ammonia oxidation [66, 67].

The uniqueness of G2 (Grass-based compost at 42 days of composting) in the heatmap is attributable to the higher abundance of pathways responsible for aromatic compound degradation and the lower Nucleoside and nucleotide biosynthetic pathways compared to other samples. The pathways for aromatic compound degradation are associated with *Gammaproteobacteria*, the most abundant phyla in G2. *Gammaproteobacteria* is responsible for aromatic compound degradation through anaerobic peripheral pathways [68].

We observed that Lantana-based compost had the highest abundance of Carbohydrate biosynthesis pathways. Such pathways include the Calvin-Benson-Bassham cycle and reductive acetyl-coenzyme A pathway [69]. These pathways predominantly utilize diverse carbohydrate metabolites in compost for microbial cellular energy needs or for building stable materials such as carboxylates. Therefore, Lantana-based compost could have had diverse, complex materials producing the metabolites necessary for the biosynthesis of stable organic forms. Furthermore, this points to the treatment as superior in the sequestration of volatile carbohydrate compounds like CO_2_ to produce readily available substrates such as acetate [70, 71]. The higher number of carbohydrate biosynthesis sequences points to that period of composting as having the best biogeochemical conditions for the assimilation of carbohydrate metabolites to produce stable organic substrates like acetate.

A similar scenario in carbohydrate degradation is a complementary indication of the presence of complex carbon polymers in Lantana-based compost compared to other treatments. Therefore, more pathways are necessary to convert complex materials such as lignin into simple carbohydrates [72, 73]. The significantly higher abundance of Carbohydrate degradation pathways at 42 days compared to other composting days indicates colonization of the compost heap by prokaryotes with high degradative capabilities of recalcitrant carbohydrates such as lignin. Such prokaryotes include Pseudomonas [59].

We did not observe significant differences in nitrogen biosynthesis pathways among the compost treatments and composting days. Nonetheless, Lantana-based Compost (L) had the most abundant pathways of nitrogen biosynthesis, pointing to the complexity of the nitrogenous content of Lantana compared to other composting materials, therefore requiring more metabolic routes before Nitrogen is in available form. The high abundance of nitrogen biosynthesis pathways in Lantana-based Compost (L) explains the higher total Nitrogen than other compost treatments. We observed a higher abundance of Nitrogen biosynthetic pathways at 63 days of composting compared to other composting days. This pathway abundance is related to the higher abundance of class *Polyangia* at 63 days in all composting treatments than on other composting days. *Polyangia* is *Myxobacteria* that utilize complex inorganic Nitrogen, biodegrading cellulolytic material [74, 75]. This period also coincides with the period when bacteria have fully utilized all available forms of carbohydrates, leaving complex materials like lignin and cellulose that are degradable by categories like *Polyangia* with the metabolic capability to break down these materials.

## Conclusion

The findings of this study show that various sources of green material have different effects on the succession of nutrients and the structure of prokaryotic communities during composting. Lantana enhances microbial diversity in compost compared to other materials but reduces microbial abundances, indicating its inhibitory property on specific categories of compost prokaryotic communities. The diverse prokaryotic taxa in Lantana-based Compost implies that due to the complexity of this material compared to others, different categories of prokaryotes are required during the metabolism of Lantana into stable and plant-useful products.

Despite the differences in earlier days, the similarity of amounts of nutrients, such as total Nitrogen, among the treatments at the end of the composting process points to composting days as influencing nutrient stability during composting. This study recommends further studies to minimize nutrient losses during composting by adding inert material such as biochar.

## Supplementary Information

**Additional file 1: Supplementary Table 1.** High-quality reads obtained from raw 16S rDNA reads after demultiplexing, quality filtering, denoising, and chimera removal.

**Additional file 2: Supplementary Figure 1.** Sequence length of resultant ASVs from the four compost regimens. 99% of the ASV had an amplicon length of about 250 bp. CSV tables have been edited to include table titles.

**Additional file 3: Supplementary Table 2.** Total ASVs recovered from different compost treatments at different sampling times. G1, L1, LTG1, T1; G2, L2, LTG2, T2; G3, L3, LTG3, T3;G4, L4, LTG4, T4.

**Additional file 4: Supplementary Table 3.** Shifts in abundances of prokaryotes within various compost treatments along composting days.

## Data Availability

The 16S rRNA gene sequences of total compost biomes used in this manuscript have been submitted to the NCBI. The accession number is PRJNA822850.
