# Supplementary figures and images for: Composition, structure, and functional shifts of prokaryotic communities in response to co-composting of various nitrogenous green feedstocks
Source: BMC Microbiol. 2023 Mar 2;23:50. doi: 10.1186/s12866-023-02798-w (PMC9979578; doi:10.1186/s12866-023-02798-w)

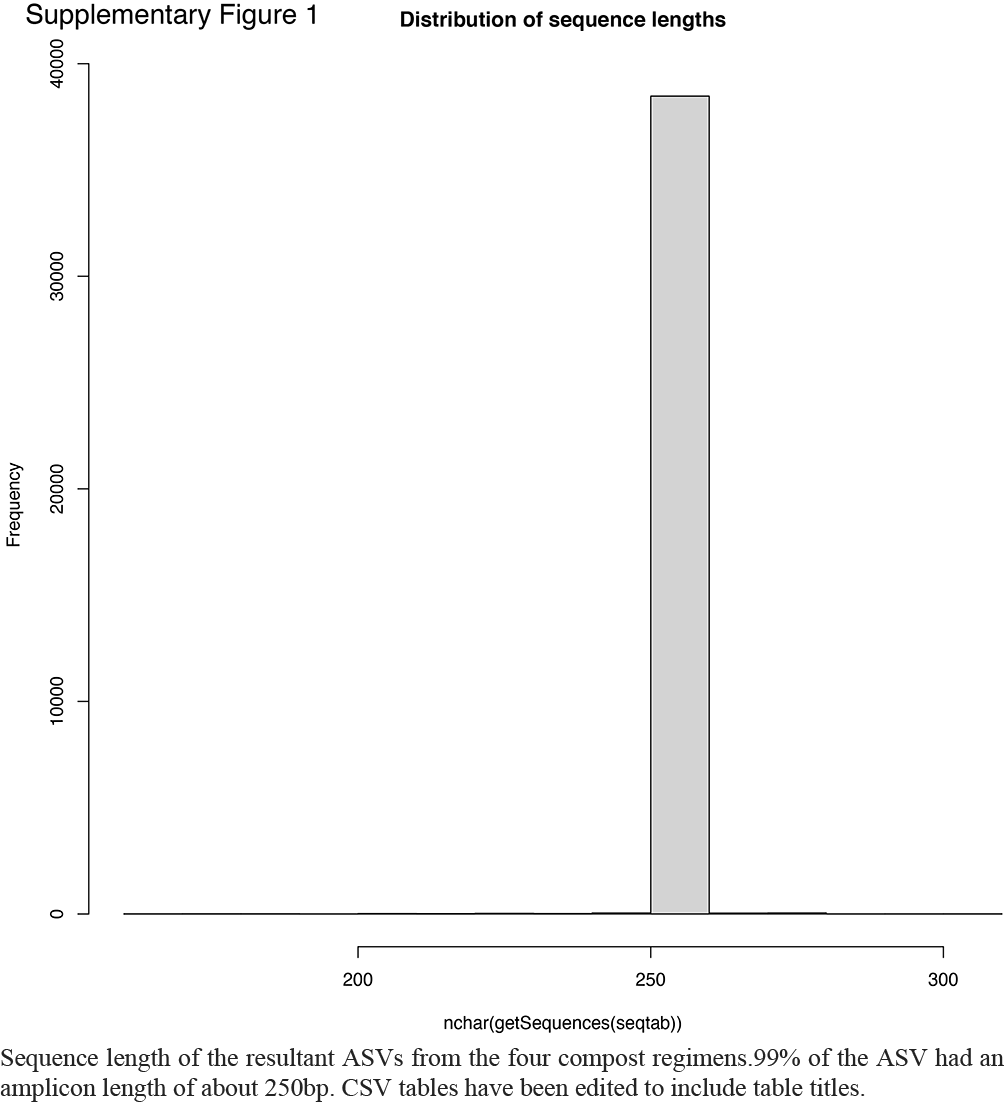

Supplement: Supplementary file 2 — Additional file 2: Supplementary Figure 1. Sequence length of resultant ASVs from the four compost regimens. 99% of the ASV had an amplicon length of about 250 bp. CSV tables have been edited to include table titles. [file 12866_2023_2798_MOESM2_ESM.tif]
